# Utilization of Insecticide Treated Bed Nets (ITNs) among Caregivers of Children under Five Years in the Ho Municipality

**DOI:** 10.1155/2019/3693450

**Published:** 2019-04-01

**Authors:** Kennedy Diema Konlan, Milipaak Japiong, Kennedy Dodam Konlan, Agani Afaya, Solomon Mohammed Salia, Joseph M. Kombat

**Affiliations:** ^1^Department of Public Health Nursing, School of Nursing and Midwifery, University of Health and Allied Sciences, PMB31, Ho, Ghana; ^2^Department of Nursing, School of Nursing and Midwifery, University of Health and Allied Sciences, PMB31, Ho, Ghana; ^3^Department of Nursing, West End University College, Accra, Ghana; ^4^Department of Paediatrics, School of Allied Health Sciences, University for Development Studies, Tamale, Ghana

## Abstract

**Background:**

In Ghana, attempts to control malaria through antimalarial medications are currently threatened by the emergence and spread of drug resistant malaria parasites. This, together with the increasing incidence of malaria, has heightened the need for a more effective method of controlling the spread. The use of Insecticide Treated Bed Net (ITN) has been recognised as an effective measure in the prevention of malaria.

**Objective/Purpose:**

In this study, we examined the utilisation of ITN among caregivers of children under five years in Ho municipality of Ghana.

**Methods:**

This descriptive cross-sectional study recruited 283 household representatives through a multistage sampling method. A questionnaire was used to collect the data and was analyzed using STATA version 14. Descriptive and inferential statistics were adopted in presenting the data.

**Results:**

Ownership of ITN was higher (80.7%) than its utilisation (41.7%). The age of caregiver was strongly associated with the utilisation of ITN (AOR=2.00, 95% CI=0.00, 0.02, p<0.001) among children less than five years. Caregivers aged 26-35 were 49% times less likely to use an ITN as compared to those aged between 17 and 25 and the difference was statistically significant.

**Conclusion:**

In order to ensure a high ITN coverage and utilisation, there is the need for continuous distribution of ITNs to households. Households should be sensitized to use the nets to prevent the continuous spread of malaria.

## 1. Introduction

Attempts to prevent malaria through antimalarial medications and insecticides are threatened by the emergence and spread of drug resistant malaria parasites and insecticide resistant vector mosquitoes [1, 2]. This together with the increasing incidence of the disease heightened the need for a more effective method of preventing the disease [2], malaria. Malaria still remain a major problem in Ghana especially among children under five years as Mba and Aboh, 2017, reported that overwhelming majority of the in-patients (41.5%) are children aged 0 – 4 years, while the number of the in-patient cases generally decreases with advancing age [3]. Case management is inefficient in the presence of drug resistance and control strategies heavily depend on vector control in such settings. ITNs are one of the effective tools in the prevention of malaria [4]. This great tool has been proven to be highly effective in all parts of the world as an effective method of reducing man–vector contact and child morbidity and mortality [5].

The use of ITN is feasible in the context because plain nets can easily be converted into ITN. Insecticide treated nets are nets that are made from cotton, polyethylene, polyester, polypropylene, or nylon and are treated with a special chemical called pyrethroid insecticide [6]. WHO is still committing fund towards the programme to ensure that targets in malaria morbidity and mortality reduction are achieved. In 2013, international and domestic funding for malaria control and elimination totaled US$ 2.7 billion [7].

There exists now a strengthened commitment of the global community to scaling up ITN distribution to vulnerable groups in malaria endemic areas [8]. Since 2002, African countries have been scaling up free or highly subsidized distribution of ITN to under five children and pregnant women in rural areas [9]. As a result, there has been a substantial increase in mosquito net coverage in African countries. The World Health Organization (WHO) recommends that in malaria endemic areas, all pregnant women should receive malaria chemoprophylaxis and sleep under ITNs [9]. Roll Back Malaria initiation in African countries increased access to chemoprophylaxis and use of ITNs by pregnant women and children under five years [10] that have become an integral part of malaria control strategies in Ghana and Africa at large.

The 2015 goals of the World Health Organization's (WHO's) Roll Back Malaria Partnership are to reduce global malaria cases by 75% and to reduce malaria deaths to near zero through universal coverage by effective prevention and treatment interventions. Among other preventive interventions, WHO recommends the use of insecticide treated nets (ITNs), particularly Long-Lasting Insecticide Nets, which have been shown to be cost-effective, to reduce malaria episodes among children under 5 years of age by approximately 50% and all-cause mortality by 17% [11]. Universal coverage with ITNs is defined as use by > 80% of individuals in populations at risk [11].

The use of ITNs is largely affected by the knowledge of people [12]. Behavioral patterns of people-utilization of the ITN are dependent on their knowledge on the consequence of nonuse [12]. Researchers give varied indications on the use of the ITN and peoples level of knowledge. Toe et al., 2009, reported that despite evidence that the use of ITNs decreases malaria-related morbidity and mortality, the use of ITNs in Africa remains relatively low [13]. Estimates suggest that in 2005, only 3% of children under five years of age slept under ITNs, while up to ten times as many are thought to sleep under any bed net [13]. This shows that the fact that ITNs are very effective in malaria prevention does not necessarily mean that people will use them after they have received them [12]. While the evidence based on the effectiveness of ITNs in reducing malaria transmission has grown rapidly in recent years, utilisation rates of ITNs in most African countries have been very low [8].

In Ghana, although there are several malaria control strategies, the emphasis is on the use of ITNs as the major control strategy by program managers [14]. Programme managers place specific emphasis on children under five and pregnant women because of the increased susceptibility to the disease. Azabre, Teye, and Yaro [14], however, showed that many households were not using this strategy because of poverty, inconvenience, and the belief that the strategy is not effective for controlling malaria [14]. Most people rather use traditional malaria control strategies, including drinking of herbs and avoiding sweets which have no direct effect in controlling malaria [14]. However, in a study conducted in the Hohoe municipality of the Volta region of Ghana, ITN use among respondents was 76.6% [15].

Despite the measures to curb malaria, the disease still remains one of the leading causes of death, especially among children under five years of age in Ghana [8] and in the Ho municipality. Axame et al., 2016, reported that in the Ho municipality, ownership of LLIN was high but utilization was very low. Ownership of LLIN was 81.4% while usage was 42.5%. Level of education significantly influenced LLIN ownership (p=0.003) and utilization (0.020) [16]. Volta region has the highest prevalence of malaria in the country [3]. Mba and Aboh, 2017, showed that malaria cases are prevalent in the Jasikan, Hohoe, Kpando, Ho, and Keta districts. Districts that lie within the middle and southern belts enjoy two rainy seasons that are conducive to the vector that causes malaria.

It is for this reason that this study sought to describe the use of ITNs among caregivers of children under five years in the Ho municipality. The finding of this study is a source of information to the District Health Management Team (DHMT), NGOs, government, and private enterprises who are involved in the promotion of ITNs in the fight against malaria among children. This study can be useful to the policy makers, the Ministry of Health (MoH) specifically in the department of malaria control.

## 2. Methods

### 2.1. Study Setting

The Ho municipality is one of the 25 administrative districts/municipalities in the Volta region of Ghana. It is located between latitudes 6°20′′N and 6°55′′N and longitudes 0°12′E and 0°53′E [17, 18]. The Municipality shares boundaries with Adaklu and Agotime-Ziope districts to the south, Ho West district to the north and west, and the Republic of Togo to the east. Its total land area is 2,361 square kilometers thus representing 11.5% of the Volta region's total land area [17, 18].

According to the 2010 population and housing census, the population of the Ho municipality was 177,281 which represented 8.4% of the Volta region's total population in 2010 [17, 18]. About 62% percent of the population reside in urban areas [17, 18]. The youth population in the municipality accounts for 31% of the population with a small number of elderly persons (population aged 65 years and older). The total age dependency ratio for the municipality is 59.0 [17, 18]. The municipality has a household population of 172,068 with a total number of 49,826 households. The average household size of the municipality is 3.45 persons. Children constitute the largest proportion of households and accounts for 34.1% [17, 18].

The Total Fertility Rate (TFR) for the municipality is 2.6 and the General Fertility Rate (GFR) is 74.4 births per 1000 women aged 15-49 years. There are three major government health facilities within the municipality that provide curative, preventive, and therapeutic services. These are the Volta Regional Hospital, Ho Municipal Hospital, and Ho Polyclinic. There are other private health facilities that serve the municipality to support the services of these three [17, 18].

The general relief of the municipality is made up of both mountainous and lowland areas. The mountainous areas are mostly to the north and northeast which are part of the Akuapem - Togo Range and have heights between 183 and 853 metres tall. The lowland areas are to the South of the municipality and are between 60 and 152 metres in height [17, 18]. The general drainage pattern is southwards and dominated by rivers like Tsawe (Alabo) and Kalapa, which flow into the lower Volta or Avu Lagoon. The rainfall pattern is characterized by two rainy seasons referred to as the major and the minor seasons. The major season begins from March to June while the minor season is from July to November [17, 18].

### 2.2. Study Design, Population, and Sampling

The study adopted descriptive cross-sectional study design. The study engaged caregivers of children under five years from selected households in selected communities in the Ho municipality because children under five years are at an increased risk to malaria with most programme interventions targeted at them. The total number of people living in the municipality is 177281 [17, 18]. Children under five years constituted 11.6% (19618) of the entire population [17, 18].

A multistage sampling technique was used to select respondents. First the district was zoned into subdistricts and communities selected using simple random sampling. The names of all communities were listed to form a sampling frame of clusters. Using lottery method of simple random sampling without replacement, 2 persons were blinded and randomly made to select a piece of paper from each submunicipality (2 communities per submunicipality). Respondents within each community were selected using convenience sampling method. For each chosen community, caregivers with children under 5 years were made to respond to a questionnaire upon consent. Proportionate sampling method was used to select the required number from each community.

The minimum sample size required for this study was 284 households with children aged under five years. The formula by Degu and Tessema [19] was used for the sample size estimation [19]:

(1) $$\begin{array}{c} N=\frac{{Z_{\mathrm{\alpha /}2}}^{2}P\left(1-P\right)}{e^{2}} \end{array}$$

where N = minimum sample size required, Z = Z score (reliability coefficient) of 1.96 at 95% confidence interval, P = prevalence which is 77.0%=0.77 (77.0% prevalence on usage of long lasting insecticide treated nets) [15] in Hohoe municipality among mothers with children under five years), and e = margin of error of 5%. The sample size of N =272 was obtained. Adjusting for non-response rate of 5% gives the total sample size of 284.

### 2.3. Pretest

Pretesting of the questionnaire was done in the Wumenu community in the Adaklu district. This tested the validity and reliability of the instrument. The pretest provided an opportunity to rephrase some of the items to reduce ambiguity and replace some misplaced ones.

### 2.4. Data Collection

We secured ethical clearance from the Institute of Health Research of the University of Health and Allied Sciences research and ethics committee. The researchers sought permission from the municipal assembly through the municipal health management team to conduct the study within the municipality. The researcher explained to respondents the need to participate in this study. The researchers were also informed that participation in this study is purely voluntary.

Caregivers of children under five completed or were assisted to complete a questionnaire. The questionnaire was divided into four main sections (A-D). Section A of the questionnaire was on demographic data. Section B focused on knowledge on ITN use. Section C concentrated on the proportion of ownership and use of ITN. Section D is dedicated to assessing the factors that affect use of ITN among caregivers of children under five years.

### 2.5. Data Analysis

Data was coded, entered into Microsoft Excel 2013, and cleaned. The data were later transported into STATA statistical software Version 14 for analysis. Descriptive and inferential statistics were adopted in analyzing the data. While the descriptive statistics comprised frequencies, percentages, mean, and standard deviation, the inferential statistic used was logistic regression. Results of the regression analysis were calculated based on 95% confidence level (alpha= 0.05) and tests using measures of association such as odds ratio. A p-value less than 0.05 was considered statistically significant. Utilization was defined as when study subjects responded to the affirmative having slept under the ITN in the night preceding the study.

### 2.6. Ethical Considerations

Ethical clearance was acquired from the University of Health and Allied Sciences, Institute of Health Research's research and ethics Committee (UHAS REC A.3 [24} 17-18). Formal permission for the study was obtained from the Ho Municipal Health Directorate and Community leaders. A written informed consent was obtained from all participants. The caregivers of children under five were assured that findings from the study and its dissemination will not have their names or any information that can be used to trace them. Special codes were assigned to participants. This was to ensure that individuals were not identified by their names. Individuals were aware that they could change their mind from participating in the study and could leave freely without any harm.

## 3. Results

### 3.1. Sociodemographic Characteristics

The mean age of children was 3.1 ± 1.7 in the households surveyed and the mean age of caregivers was 33.3 ± 9.2. Out of 283 caregivers, those aged between 17 and 25 years were 18.0%, 26-35 years constituted 45.2%, and those 36 years and above formed 36.75%. The majority (35.7%) of the caregivers attained tertiary level of education while those who had no education were 12.7%. Most (92.6%) of the respondents were Christians and 64.3% were married at the time of the survey (Table 1).

### 3.2. Knowledge of Respondents on ITN

The majority (90.8%) of the respondents indicated that ITN is key in the prevention of Malaria. Some respondents (20, 7.1%) also indicated that for the ITN to be effective, it has to be air dried frequently. The majority (80.6%) owned ITNs and 41.7% of them slept under the ITNs with their children the night before the survey. The findings showed 88.2% of caregivers aged 17-25 owned an ITN and 54.9% of them slept under the ITN the night before the survey. Most (75.8%) of the caregivers aged 26-35 also owned an ITN and 38.3% slept under the ITN the night before the survey. Among caregivers aged 36 and above, 82.7% owned an ITN and 39.4% of them slept in it the night before the survey.

Caregivers with junior high school level of education who owned an ITN constituted 1.2%. Out of this 48.5% slept under ITN the night preceding the survey. Also, 92.0% of caregivers with senior high school level of education owned an ITN and 46.0% slept under them the night preceding the survey. Out of caregivers who have no educational background, 58.3% owned ITNs and 33.3% slept under the ITN the night before the survey.

The majority (85.7%) of married caregivers owned an ITN and 38.5% slept under it the night before the survey. Again, 77.6% of single caregivers owned an ITN and 44.8% slept under it the night preceding the survey. Only 22.2% of caregivers who were cohabiting owned an ITN and none slept under it the night preceding the study. The majority (82.1%) of the caregivers who were Christians owned an ITN at the time of the survey and 43.5% slept under an ITN the night preceding the study as 68.4% of Muslim caregivers owned an ITN and 21.1% slept in it the night before the study. About 80% of employed caregivers owned an ITN and 39.7% slept under it the night before the study. About 85% of unemployed caregivers also owned an ITN and 61.5% slept under the ITNs the night preceding the study.

### 3.3. Factors Associated with ITN Utilization

As shown in Table 2, caregivers aged between 26 and 35 years were 2 times more likely to use an ITN as compared to the other age groups and there was a strong association (AOR=2.00, 95%CI=0.00,0.02, p<0.001). Caregivers with JHS education were 1.2 times more likely to use an ITN as compared to the others (AOR=1.16, 95%CI=0.91, 1.49, p=0.240). Employed caregivers were also 1.51 times more likely to use an ITN than unemployed caregivers and there was no association (AOR=1.51, 95%CI=0.47, 4.92, p=0.490).

Married and separated caregivers were 5.50 and 4.96 times more likely to use an ITN (AOR=5.50, 95%CI=0.15, 200.76, p=0.353) and (AOR=4.96, 95%CI=0.60, 41.24, p=0.139), respectively. Caregivers who responded that ITNs have side effects were 49% times less likely to use an ITN as compared to those who responded otherwise and caregivers who said ITNs were expensive were 1.34 times more likely to use an ITN as compared to those who said ITNs were note expensive (AOR=1.34, 95%CI=0.50,3.60, p=0.552). Caregivers having a household number between 5 and 10 were 1.82 times more likely to use and ITN as compared to those having a household number between 1 and 4 (AOR= 1.82, 95%CI=0.44,7.44, p=0.402).

## 4. Discussion

This study assessed the utilization of the ITN among caregivers of children under five in the Ho municipality. Utilization was defined as when study subjects responded to the affirmative having slept under the ITN in the night preceding the study. Preventing malaria is the prime focus of all stakeholders in the malaria control programme and as such caregivers are supposed to have adequate knowledge on the use of the ITN. Caregivers were 59% times less likely to use ITN with increased knowledge on malaria and the use of the ITN. With this heightened knowledge on malaria and ITN usage, there is increasing likelihood caregivers will use alternative means of preventing malaria than using solely the ITN. The more the caregivers were educated on the risk factors of malaria and the significance of net use, the more these study participants practiced good environmental hygiene thus reducing the utilization of the ITN. But respondents who had less knowledge on ITN and malaria tend to use the ITN more frequently.

Ownership of ITN among caregivers with children under five in this study was found to be 80.6%. This result is similar to studies conducted in highlands of Western Kenya which revealed that ownership during the dry season was 73.8% [20]. This is also in line with the study conducted by Nyavor et al. [21] in the Hohoe municipality, Ghana, and Wanzira et al. [22] in Central Uganda, which showed that ownership of ITN was 81.3% and 84%, respectively. The high ownership of ITN in this study could be attributed to the free distribution of ITNs to mothers during pregnancy and at Child-Welfare Clinics [21, 22].

In this current study, utilization of ITN was 41.7% among children under five in the Ho municipality. This was lower than what was reported by Ghana Demographic Health Survey (GDHS) where ITN use among children under five was 59% [23]. This study was conducted within the major rainy season [17, 18] of the municipality with an increased likelihood of transmission of the plasmodium. Other studies Aung et al. [24] and Aderibigbe et al. [25] have also reported increased utilization of ITNs. It was revealed that not all those who own nets use them [23]. Also in line with studies carried out in Harari National Regional State, Eastern Ethiopia showed that ITN utilization was 73.3% [26]. The low percentage of ITN utilization in this study could be attributed to the season in which the study was conducted, which was a dry season.

Caregivers aged 26-35 were 49% times less likely to use an ITN as compared to those aged between 17 and 25 and the difference was statistically significant. Also, caregivers aged 36 and above were 46% times less likely to use an ITN as compared to those aged 17-25 and the difference was not statistically significant. Education on the use of ITN is intensified for women who are primids. Women aged 17-25 years are largely primi-para and had to receive intense education and care during antenatal care services. This special attention most likely to be attributed to this age category may be the reason they have high chance of using ITN compared to those 25 to 35 years who might not be primi-para.

There was an association between caregivers with tertiary level of education and the utilization of ITNs. Caregivers with tertiary level of education were 53% times less likely to use an ITN as compared to those with no educational background and the difference was statistically significant. The general consensus is that with increasing education, most women are likely to engage in activities that decrease their risk of contracting infectious diseases especially in pregnancy. Axame et al., 2016, reported that level of education significantly influenced LLIN ownership (p=0.003) and utilization (0.020) [16].

Also, employed caregivers were 58% times less likely to use an ITN as compared to their unemployed colleagues, and the difference was statistically significant. There was an association between caregivers who were separated and the utilization of ITNs. Caregivers who were separated were 78 times more likely to use an ITN as compared to those who were cohabiting and the difference was statistically significant. Separated caregivers may tend to be more conscious of the health of their child compared to the couples who were staying together.

There was an association between ITN side effects and ITN utilization. Caregivers who responded that ITN has side effects were 73% times less likely to use an ITN as compared to those who said ITN has no side effects and the difference was statistically significant. The level of knowledge and understanding of the people of the efficacy of a particular intervention has an influence on the way they eventually execute their behavior. The practices of a group of people is largely influenced by their level of knowledge and the belief that a particular behavior will yield desired results that is less injurious to the individual [12]. Evidence from some parts of Ghana has shown that over 40% of ITNs available in the households go unused. This could erode the gain made in ITN use over the years [27].

Also, with an increasing perception of the cost involved in the use of the ITN, community members are likely to use the ITN because of the value they place in acquiring one. Caregivers who said ITNs were expensive were 33% times less likely to use an ITN as compared to those who said it was not expensive and the difference was not statistically significant.

The results of this study serve as a useful insight to the use of ITN among caregivers of children under five years in the Ho municipality. As using these findings may help in policy formulation, its immediate use must be done with caution. This study assessed respondent self-reporting scheme with a higher likelihood that responses were most likely going to be positive especially with the heighten education on the use of the ITN in major media outlets in Ghana. Also, to be able to determine the trends of utilization of the ITN over time, the observation and/or longitudinal study methods may be adopted to assess the pattern of use of the ITN and its resultant impact on preventing malaria in children under five years.

## 5. Conclusion

The study showed that utilization of ITN was low (41.7%). It was revealed that not all those who own nets use them. The factors that influence caregivers of children under five years in the use of ITNs include, caregivers age, caregiver's level of education, employment status of caregiver, marital status of caregiver, religion of caregiver, side effects of ITN use, and also the price perception of an ITN.

There should be a continual free distribution of the ITN at ANCs, health centers, and through campaign strategies by health workers in order to reach out to the entire population. Local government authorities must be interested in supporting strategies that will increase ownership of the ITN among the at-risk population while the continual and consistent utilization is enhanced through education. This could be done by targeting the pregnant mothers and the children under 5 in the district since they are the most vulnerable to the malaria disease.

In order to appreciate the use of ITN, it would be required that more health education is done by the Ministry of Health/Ghana Health Service to enable better understanding on the ITN and how it works. The education must target certain demographic segment of the population as utilization and level of knowledge among the various demographic characteristics varied.

## Figures and Tables

**Table 1:** Socio-demographic characteristics of respondents.

Variables	Attributes	Frequency (N=283)	Percentage (%)
*Mean Age care givers (SD)*= 33.3 (9.2) N=283			

Age	17-25	51	18.0
	26-35	128	45.2
	36+	104	36.7

Educational level	None	36	12.7
	Primary	30	10.6
	JHS	66	23.3
	SHS	50	17.7
	Tertiary	101	35.7

Marital status	Never married	67	23.7
	Co-habiting	9	3.2
	Married	182	64.3
	Separated	22	7.8
	Divorced	3	1.1

Religion	Christian	262	92.6
	Muslim	19	6.7
	Other	2	0.7

**Table 2:** Factors associated with ITN utilization.

	Variables	Frequency [N=260] N(%)	COR (95%) p-value	AOR(95%) p-value
Age distribution	17-25	Ref.		
	26-35	49(32.3)	0.51(0.27,0.98)0.045	2.00(0.00,0.02)<0.001
	36 and above	41(39.4)	0.54(0.28,1.06)0.071	

Education status	None	Ref.		
	JHS	32(48.5)	0.54(0.24,1.25)0.149	1.16(0.91,1.49)0.240
	Primary	20(66.7)	2.07(0.86,5.02)0.106	
	SHS	23(46.0)	0.91(0.44,1.88)0.794	
	Tertiary	31(30.7)	0.47(0.25,0.90)0.022^*∗*^	

Employment status	Unemployed	Ref.		
	Employed	102(39.7)	0.42(0.19,0.95)0.036^*∗*^	1.51(0.47,4.92)0.490

Marital status	Co-habiting	Ref.		
	Married	70(38.5)	11.91(0.68,207.78)0.090	5.50(0.15,200.76)0.353
	Separated	18(81.82)	78.11(3.79,1608.07)0.005^*∗∗*^	4.96(0.60,41.24)0.139
	Single	30(44.8)	15.45(0.86,276.31)0.063	0.60(0.47,7.69)0.698

Religion	Christian	Ref.		
	Moslem	4(21.0)	0.38(0.13,1.11)0.076	0.43(0.13,1.44)0.173

Awareness of ITN use	No	Ref.		
	Yes	278(98.2)	10.50(3.87,28.54)<0.001^*∗∗*^	0.41(0.13,1.23)0.118

Household size	1-4	Ref.		
	5-10	11(35.5)	0.76(0.36,1.63)0.480	1.82(0.44,7.44)0.402

Awareness of ITN side effects	No	Ref.		
	Yes	64(33.7)	0.37(0.22,0.61)<0.001	0.51(0.19,1.37)0.180

ITN too costly	No	Ref.		
	Yes	25(36.8)	0.77(0.44,1.34)0.354	1.34(0.50,3.60)0.552

## Data Availability

The data used to support the findings of this study are included within the article.
